# The effects of circulating proteins and metabolites on diabetic foot ulcer: A Mendelian randomization study and mediation analysis

**DOI:** 10.1097/MD.0000000000048581

**Published:** 2026-05-08

**Authors:** Minghao Wang, Lei Huang, Xin Hu, Hu Yang, Shenghui Yu

**Affiliations:** a Department of Emergency Medicine, Chengdu Integrated TCM and Western Medicine Hospital, Chengdu, Sichuan, P.R. China; b Department of Gastroenterology, Chengdu Integrated TCM and Western Medicine Hospital, Chengdu, Sichuan, P.R. China.

**Keywords:** causal relationships, circulating proteins, diabetic foot ulcer, Mendelian randomization, metabolites

## Abstract

Diabetic foot ulcers (DFUs) are a common and severe complication of diabetes, and understanding the biological mechanisms underlying their development is crucial for identifying potential biomarkers and therapeutic targets. Recent advances in Mendelian randomization (MR) and mediation analysis have provided new ways to explore the causal relationships between circulating proteins, metabolites, and diseases. This study aims to investigate the causal relationships between circulating proteins, metabolites, and DFU using MR and mediation analysis, leveraging publicly available genome-wide association studies datasets. We applied a bi-directional 2-sample MR approach to analyze 2923 circulating proteins, 1400 metabolites, and DFU, with single nucleotide polymorphisms as instrumental variables. Mediation analysis was used to explore potential causal pathways between proteins, metabolites, and DFU. Our analysis identified 42 circulating proteins and 50 metabolites with significant unidirectional causal effects on DFU. Notably, specific proteins influenced metabolites that may, in turn, contribute to DFU development. Mediation analysis revealed a causal chain where the protein GLB1 exerts an effect on DFU through the metabolite 2-linoleoylglycerol (18:2), with a mediation ratio of 10.91%. Sensitivity analyses confirmed the robustness of our findings, with no significant bias from heterogeneity or pleiotropy. These results highlight the complex interactions between proteins, metabolites, and DFU. Our findings suggest that circulating proteins may influence DFU through specific metabolites, providing valuable insights into potential biomarkers and therapeutic targets for DFU. This study demonstrates the utility of MR and mediation analysis in understanding the biological mechanisms underlying complex diseases like DFU.

## 1. Introduction

Diabetic foot ulcer (DFU) epidemiology shows a global prevalence of approximately 6.3% among people with diabetes, with a lifetime risk of 19% to 34% of developing one.^[1]^ The 5-year mortality rate for individuals with a DFU is approximately 30%, exceeding 70% for those with a major amputation.^[2]^ While numerous studies have examined these elements separately, a full picture of how they interact in the progression of DFU is still lacking.^[3,4]^

Comprising the full spectrum of blood proteins, the circulating proteome acts as a dynamic system that mirrors an individual’s genetic background as well as environmental and lifestyle influences. Studies concentrating on selected biomarkers have revealed that combining proteomic and genetic datasets can identify causal associations between proteins and disease processes, offering promising avenues for drug discovery in the bloodstream.^[5–8]^ The value of conducting large-scale, unbiased population studies of the circulating proteome for uncovering new insights into disease biology – particularly in improving the understanding, management, and prognosis of common conditions such as acne – remains to be fully established.

In recent years, Mendelian randomization (MR) has emerged as a widely adopted analytical strategy for investigating potential causal links between diverse exposures and clinical outcomes. This approach leverages genetic variants – particularly single nucleotide polymorphisms (SNPs) – as instrumental variables (IVs) to infer the causal effect of genetically predicted exposures on genetically predicted outcomes.^[9–12]^ In comparison, most epidemiological investigations into the causal connections between DFU, the circulating proteome, and metabolites in circulation continue to depend on established approaches like cross-sectional, case-control, and cohort analyses. Nevertheless, effect estimates derived from these traditional methodologies are frequently subject to important limitations, including residual confounding and bias from reverse causation.^[12]^

Therefore, the objective of this research is to apply MR to explore the possible causal relationships between the circulating proteome and DFU, and further to assess whether metabolites act as intermediaries in linking circulating proteins with DFU onset.

## 2. Materials and methods

### 2.1. Study design

**Figure 1.**
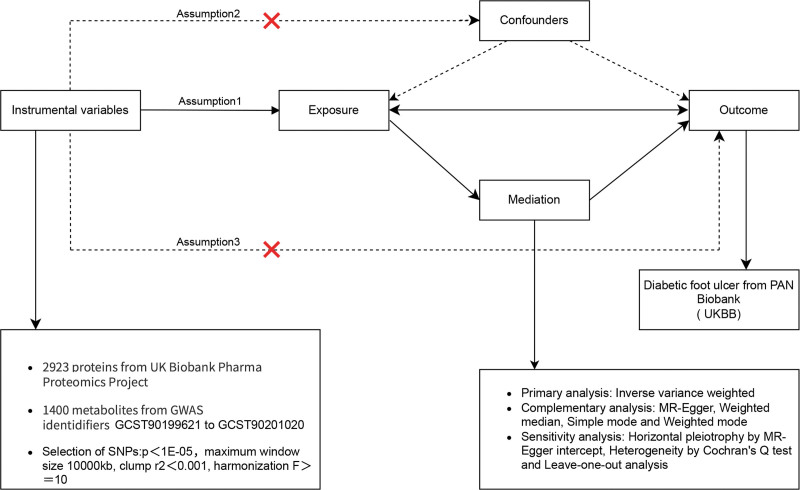
Overview of the MR Framework. A schematic representation illustrating the key assumptions of MR, including the use of SNPs as IVs to assess causal relationships between circulating proteins, metabolites, and DFU. DFU = diabetic foot ulcers, IVs = instrumental variables, MR = Mendelian randomization, SNPs = single nucleotide polymorphisms.

This analysis was conducted using publicly available datasets that had previously received approval from the relevant institutional review boards, meaning no additional ethical clearance was required. All results are presented in this article, along with supplementary materials. To assess causal relationships, we applied a bi-directional 2-sample MR approach, examining 2923 circulating proteins, 1400 metabolites, and DFU. SNPs were utilized as IVs. The study complied with the STROBE-MR guidelines. A conceptual outline of the MR assumptions is shown in Figure 1, which details the proposed causal pathways and identifies potential mediating risk factors. This diagram demonstrates the application of genetic variants as IVs and highlights the essential assumptions about causality, confounding, and pleiotropy. This framework enables rigorous testing of exposure-outcome relationships, while accounting for mediators, thus providing the foundation for the statistical methods used in this study. Further information regarding the genome-wide association studies (GWAS) and data sources is included in the supplementary tables and following sections.

### 2.2. GWAS data on DFU

Genetic summary statistics for DFU were obtained from the PAN UK Biobank (UKBB), encompassing data from 420,473 participants of European descent enrolled in the UK Biobank.^[13–15]^ Within this cohort, 130 individuals were identified as having DFU, while the remainder acted as controls. Case identification was based on thorough clinical assessments performed by trained nurses in combination with self-reported information provided by participants.

### 2.3. GWAS data on circulating proteins

Information on 2923 circulating proteins was derived from the UK Biobank (UKB) Pharma Proteomics Project, a large-scale collaboration involving 13 pharmaceutical companies aimed at generating proteomic data within a subset of the UK Biobank cohort.^[14,16]^ A total of 54,306 individuals contributed to the dataset: 46,673 (85.9%) were randomly chosen from the main cohort, 6385 (11.8%) were preselected according to consortium-defined criteria (e.g., disease phenotype or ancestry), and 1268 (2.3%) were included owing to participation in multiple COVID-19 imaging studies. After stringent quality control procedures, 53,021 participants were retained for analysis. Ethical approval for UK Biobank was granted by the North West Multicenter Research Ethics Committee, and all analyses were conducted under Application Number 93810.

### 2.4. GWAS data on serum metabolites

Summary statistics for plasma metabolomics were obtained from the GWAS Catalog, covering accession numbers GCST90199621–GCST90201020 (2023). This dataset comprised 1091 metabolites and 309 metabolite ratios from 8299 individuals of European ancestry.^[17]^ In total, GWAS data for 1400 metabolites, generated using HG19/GRCh38 reference builds, were included in this analysis and are publicly available via the EBI GWAS Catalog (https://www.ebi.ac.uk/gwas/).

### 2.5. Selection of instrumental variables

IVs in MR^[18,19]^ must satisfy 3 principal assumptions: strong relevance to the exposure, independence from confounding factors, and influence on the outcome exclusively through the exposure of interest.^[20]^ SNPs were selected according to a genome-wide significance threshold of *P* < 1 × 10^−5^ with linkage disequilibrium clumping at *r*^2^ <0.001 and a 10,000-kb window to ensure independence. To minimize bias from weak instruments, *F*-statistics were calculated for each SNP using *F* = β^2^-exposure/ SE^2^-exposure, with the proportion of variance explained (*R*^2^) derived from *R*^2^ = 2 × (1-MAF) × MAF × β^2^ SNPs with *F*-values <10 were excluded.^[21]^

### 2.6. Mediation analyses

The mutual causality between circulating proteins and DFU was assessed using 2-sample MR, expanding to include inflammatory mediators and their potential impact on DFU. After deriving MR estimates, we identified noteworthy associations (*P* <.05) using the instrumental variable approach. Next, we examined the relationships between significant components of the circulating proteins and inflammatory markers. Lastly, we conducted a preliminary evaluation of the bi-directional overall effect between circulating proteins and DFU. This effect was decomposed into mediation effects, with the indirect influences mediated through metabolites. To determine the proportion of mediation, the magnitude of the indirect effect was divided by the total effect’s magnitude.

### 2.7. Statistics

Associations between exposures and outcomes were assessed using the TwoSampleMR R package (v0.5.6) in R (v4.4.1).^[22]^ Five complementary MR approaches were applied: inverse variance-weighted random-effects model, MR-Egger regression, weighted median estimator, simple mode, and weighted mode, with inverse variance-weighted serving as the primary analytic strategy.^[23]^ Sensitivity analyses included Cochran Q test to evaluate heterogeneity,^[24]^ with results visualized using funnel plots. To detect horizontal pleiotropy, both MR-PRESSO (R package v1.0) and MR-Egger intercept tests were performed.^[25]^ SNPs identified as pleiotropic were removed before reanalysis. Additionally, leave-one-out analysis was conducted to test the robustness of findings by sequentially excluding individual SNPs. All statistical tests were 2-sided, and *P* <.05 was considered statistically significant in this exploratory setting. The total causal effect of exposures on DFU was partitioned into direct effects and indirect effects mediated by metabolites. Mediation proportions were calculated as the ratio of the indirect effect to the total effect. Confidence intervals (95%) for the indirect effect were estimated using the delta method, thereby accounting for variability in effect size estimates.

## 3. Result

### 3.1. Analysis causal associations of 2923 UKB proteins on DFU

**Figure 2.**
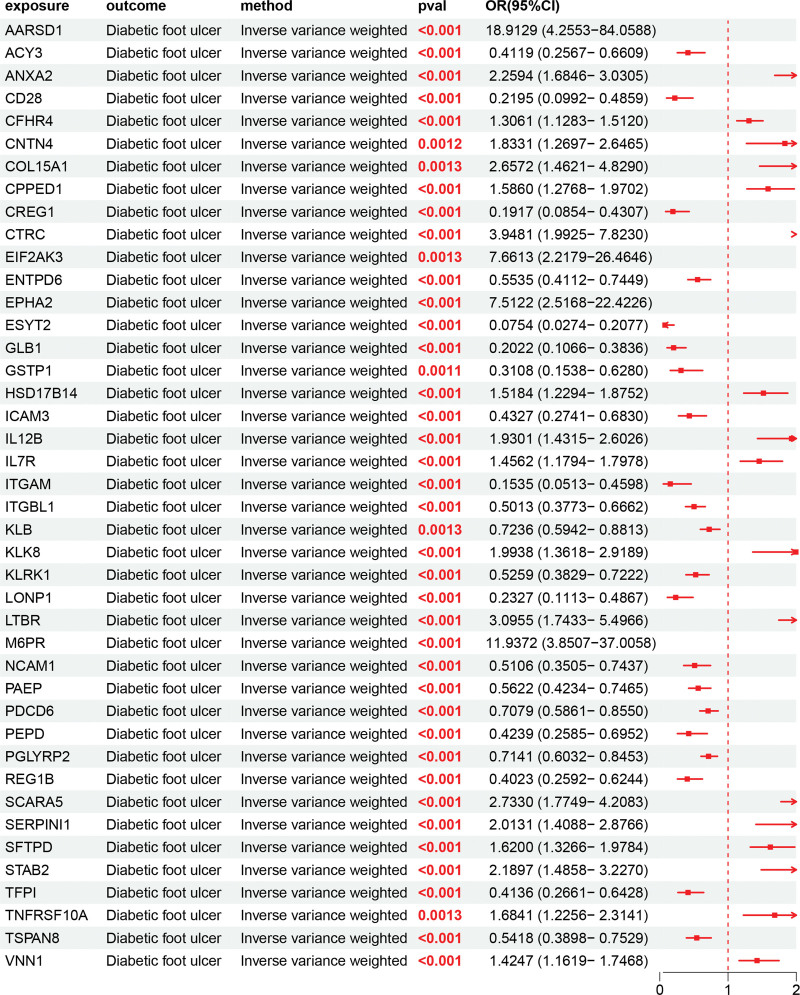
Forest plot showing the causal effects of 2923 circulating proteins on DFU obtained by inverse variance-weighted MR analysis. Each point represents an OR with a 95% confidence interval. Values pooling to the left of the vertical line indicate protective associations (OR <1), whereas those to the right indicate risk associations (OR >1). Proteins showing significant causal effects (e.g., AARSD1, EPHA2, TNFRSF10A, and IL7R) were primarily associated with pathways involved in apoptosis, vascular function, and immune regulation. AARSD1 = alanyl-tRNA synthetase domain–containing 1, DFU = diabetic foot ulcer, EPHA2 = Eph receptor A2, IL7R = interleukin-7 receptor, MR = Mendelian randomization, OR = odds ratio, TNFRSF10A = tumor necrosis factor receptor superfamily member 10A.

The causal relationships between 2923 UKB proteins and DFU were initially assessed using the bi-directional MR approach. To determine the direction of causality, we retained only the positive results where UKB proteins were considered the exposure and DFU as the outcome, excluding the scenario where DFU was the exposure and UKB proteins were the outcome. We found that 42 UKB proteins were statistically significant positive exposures relative to DFU (outcome) (Fig. 2). Among them, AARSD1 (OR = 18.91, 95% CI = 4.26–84.06), EPHA2 (OR = 7.51, 95% CI = 2.52–22.42), and LTBR (OR = 3.10, 95% CI = 1.74–5.50) showed the strongest positive causal effects on DFU risk, whereas GLB1 (OR = 0.20, 95% CI = 0.11–0.34) and ICAM3 (OR = 0.43, 95% CI = 0.27–0.68) were negatively associated, suggesting potential protective effects. The characteristics of significant SNPs with genome-wide associations (*P* <1 × 10^−5^) for exposures (UKB proteins) on outcomes (DFU) are provided in Supplementary Table S1. Heterogeneity analyses of UKB proteins on DFU are presented in Supplementary Table S2A. Horizontal pleiotropy analyses of UKB proteins on DFU are provided in Supplementary Table S2B.

### 3.2. Analysis causal associations of 1400 metabolites proteins on DFU

**Figure 3.**
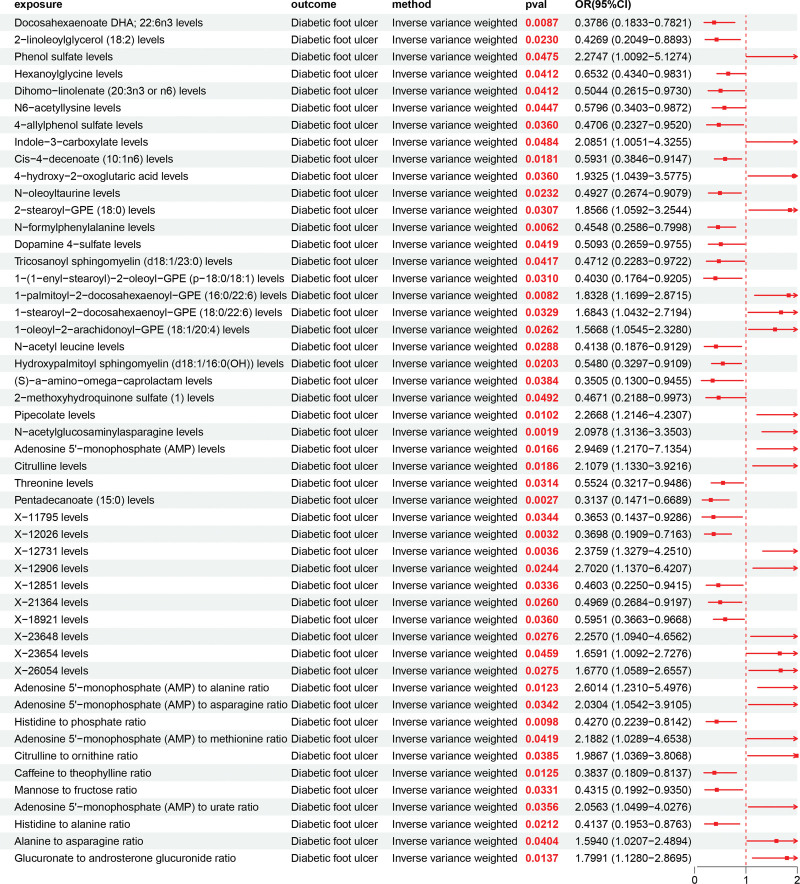
Forest plot showing the causal effects of 1400 circulating metabolites on DFU obtained by inverse variance-weighted MR analysis. Each point represents an OR with a 95% confidence interval. Values pooling to the left of the vertical line indicate protective effects (OR <1), whereas those to the right indicate risk effects (OR >1). Metabolites showing significant protective associations (e.g., DHA 22:6n3, 2-linoleoylglycerol 18:2) were primarily classified as lipid-and amino acid–related compounds. DFU = diabetic foot ulcer, DHA = docosahexaenoate, MR = Mendelian randomization, OR = odds ratio.

Similarly, we utilized the MR method to evaluate the relationship between 1400 metabolites and DFU. To elucidate causal directionality, a bi-directional MR approach was implemented, with DFU and 1400 metabolites serving as both exposure and outcome variables. We took 1400 metabolites as the exposure and DFU as the outcome for the positive results, excluding the positive results where 1400 metabolites are the outcome and DFU is the exposure. After excluding bi-directional causality, pleiotropy, and heterogeneity, 50 metabolites demonstrated unidirectional causal effects on DFU (Fig. 3). Among them, several metabolites showed significant associations: Docosahexaenoate (DHA; 22:6n3) was negatively associated with DFU risk (OR = 0.3786, 95% CI = 0.1833–0.7821), while 2-linoleoylglycerol (18:2) also exhibited a protective effect (OR = 0.4269, 95% CI = 0.2049–0.8893). In contrast, Phenol sulfate (OR = 2.747, 95% CI = 1.009–7.512) and Pipercolate (OR = 2.268, 95% CI = 1.146–4.307) were positively associated with an increased risk of DFU. These results indicate that these 3 metabolites may promote the development of DFU. The characteristics of significant SNPs with genome-wide associations (*P* <1 × 10^−5^) for exposures (metabolites) on outcomes (DFU) are provided in Supplementary Table S3. Heterogeneity analyses of metabolites on DFU are presented in Supplementary Table S4A. Horizontal pleiotropy analyses of metabolites on DFU are provided in Supplementary Table S4B.

### 3.3. Analysis causal associations of 42 UKB proteins on 50 metabolites

**Figure 4.**
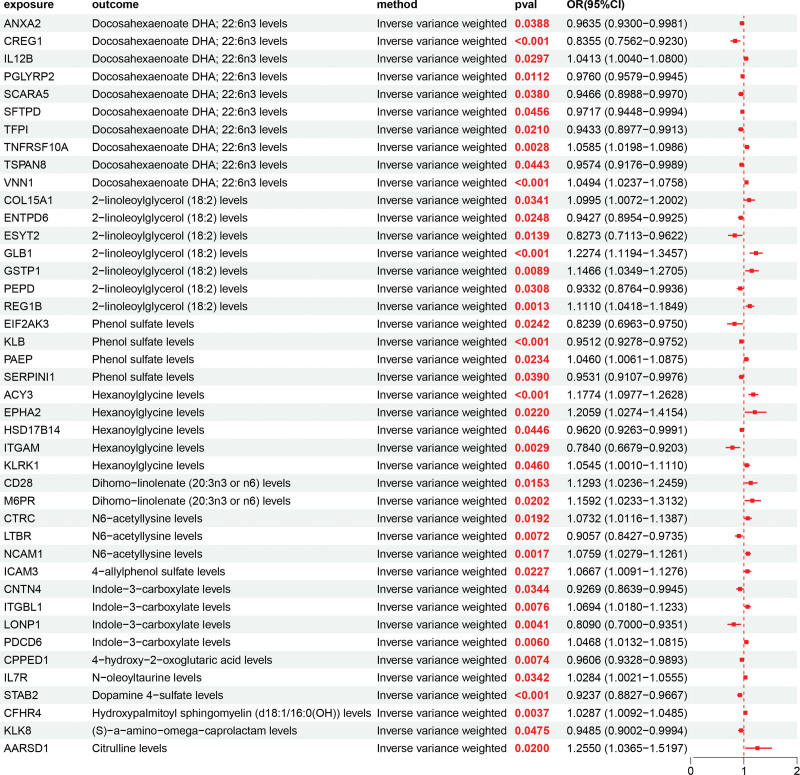
Forest plot showing the causal associations between 42 DFU-related circulating proteins and 50 significant metabolites identified by MR analysis using the inverse variance-weighted method. Each point represents an OR with a 95% confidence interval. Values pooling to the left of the vertical line indicate negative or protective associations (OR <1), while those to the right denote positive or risk associations (OR >1). Notable examples include GLB1 → 2-linoleoylglycerol (18:2) and TNFRSF10A → docosahexaenoate (22:6n3), reflecting lipid- and amino acid–related metabolic pathways potentially mediating DFU risk. DFU = diabetic foot ulcer, GLB1 = β-galactosidase (galactosidase beta 1), MR = Mendelian randomization, OR = odds ratio, TNFRSF10A = tumor necrosis factor receptor superfamily member 10A.

We investigated the impact of 42 UKB proteins relevant to DFU on 50 metabolites. To elucidate causal directionality, a bi-directional MR approach was implemented. We took 50 metabolites as the exposure and DFU as the outcome for the positive results, excluding the positive results where 50 metabolites are the outcome and DFU is the exposure. After excluding bi-directional causality, pleiotropy, and heterogeneity, we showed the significant causal association between 53 UKB proteins on 50 metabolites (Fig. 4). Among the notable associations, CREG1 showed a strong negative causal effect on docosahexaenoate (DHA; 22:6n3) levels (OR = 0.8335, 95% CI = 0.7526–0.9230), while TNFRSF10A was positively associated (OR = 1.0545, 95% CI = 1.0198–1.0896). Similarly, GLB1 demonstrated a positive effect on 2-linoleoylglycerol (18:2) (OR = 1.2274, 95% CI = 1.1194–1.3457), and KLB was positively linked with phenol sulfate (OR = 1.0460, 95% CI = 1.0061–1.0875). In contrast, EPHA2 showed a risk-increasing effect on hexanoylglycine (OR = 1.2059, 95% CI = 1.0274–1.4154). The characteristics of significant SNPs with genome-wide associations (*P* < 1 × 10^−5^) for exposures (UKB proteins) on outcomes (metabolites) are provided in Supplementary Table S5. Heterogeneity analyses of UKB proteins on metabolites are presented in Supplementary Table S6A. Horizontal pleiotropy analyses of UKB proteins on metabolites are provided in Supplementary Table S6B.

### 3.4. Scatter plots of statistically significant causal effects and leave-one-out analysis

**Figure 5.**
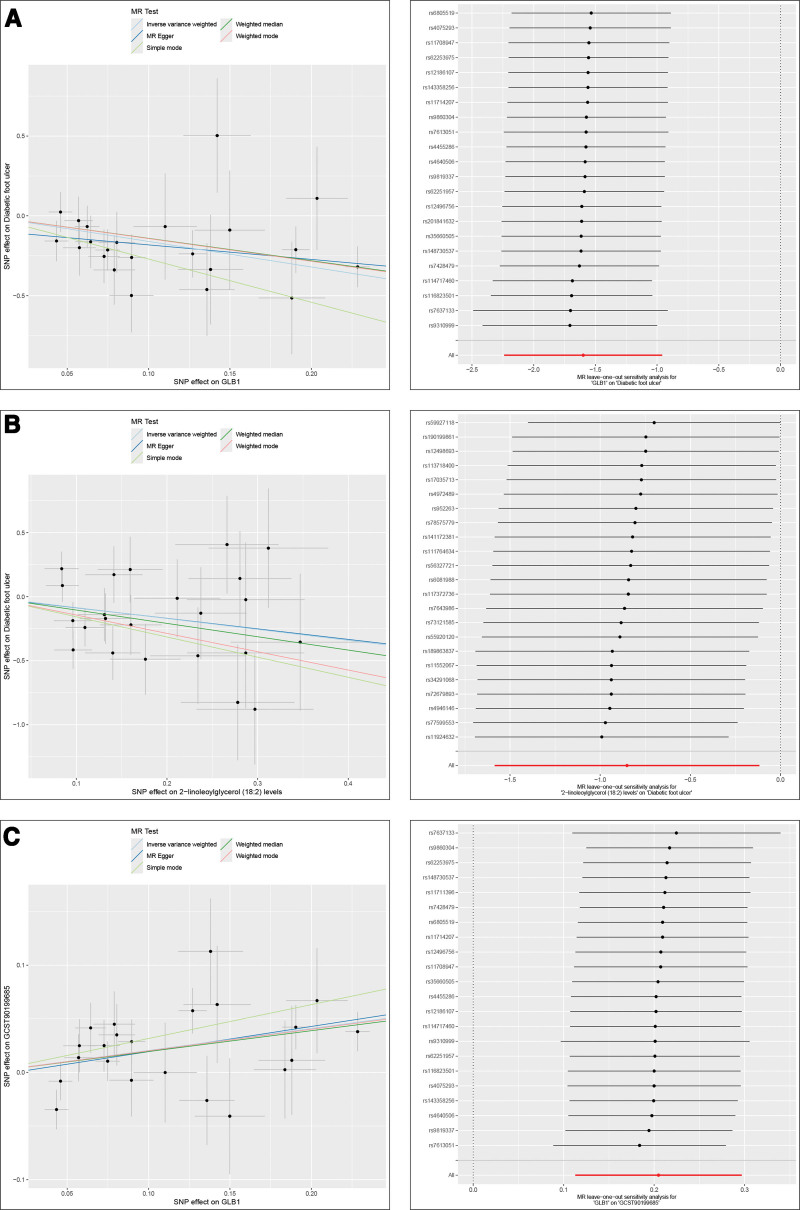
Scatter plots of statistically significant UKB Proteins associated with DFU. Scatter plots showing the significant causal effects of 42 UKB proteins on DFU identified through MR analysis. Each plot represents a protein with a unidirectional causal effect on the outcome (A). Scatter plots of statistically significant metabolites associated with DFU. Scatter plots depicting the causal effects of 50 metabolites on DFU (B). Scatter plot illustrating the causal effects of statistically significant UKB proteins on metabolites (C). DFU = diabetic foot ulcer, MR = Mendelian randomization, UKB = UK Biobank.

Our findings indicate that 42 UKB proteins and 50 disease-relatedmetabolites demonstrate causal relationships with DFU. Further investigation revealed that 42 UKB proteins (serving as exposures) exerted unidirectional causal effects on 14 metabolites (serving as outcomes). Next, we provided the scatter plots of statistically significant causal effect, and the Sensitivity analysis were performed by leave-one-out analysis (Fig. 5).

### 3.5. Analysis of mediation

**Figure 6.**
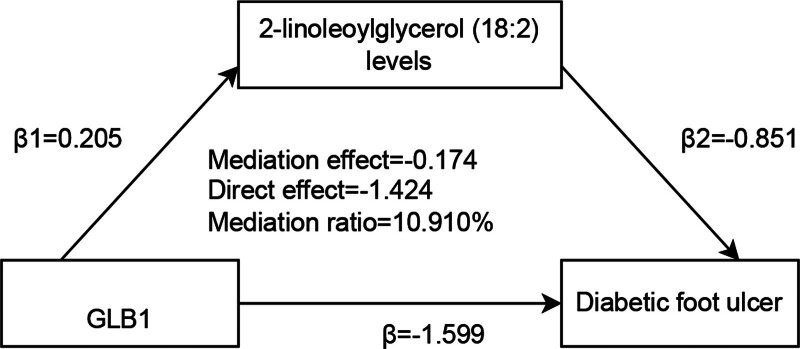
Mediation analysis of metabolites in DFU pathogenesis. This figure highlights the mediation effects of 8 metabolites in the causal pathways between 3 UKB proteins and DFU. The mediation ratios and statistical significance of the metabolites are provided. DFU = diabetic foot ulcer, UKB = UK Biobank.

Next we performed the MR mediation analysis to reveal the casual chain in UKP protein, metabolite, and DFU. The dataset presents the results of a mediation analysis investigating the relationship between exposures (GLB1), mediators [2-linoleoylglycerol (18:2) levels], and the outcome of DFU (Fig. 6). The results from Supplementary Table S7 show the mediation effects of 2-linoleoylglycerol (18:2) levels on the outcome of DFU for the exposure GLB1. The exposure effect (β1) was 0.2049, while the mediation effect (β2) was −0.8513, leading to a mediation effect of −0.1744. The lower and upper confidence intervals for the mediation effect were −0.3440 and −0.0048, respectively. The total effect was −1.5986, with a standard error of 0.0865. The mediation ratio was 10.91%, indicating that GLB1 exposure has a significant mediating effect through 2-linoleoylglycerol (18:2) levels. This suggests that the mediation contributes substantially to the negative total effect on DFU.

## 4. Discussion

In this MR study, we identified a potential causal relationship between the protein GLB1 and the risk of DFU, mediated through the metabolite 2-linoleoylglycerol (18:2). The inverse odds ratio (OR = 0.20, 95% CI = 0.11–0.38) suggests that higher genetically predicted GLB1 levels may exert a protective effect against DFU. This finding provides new insight into the molecular pathways linking lipid metabolism, inflammation, and tissue repair in diabetes complications.

GLB1 encodes β-galactosidase, a lysosomal enzyme responsible for the degradation of GM1 ganglioside. Deficiency or reduced activity of GLB1 can lead to the accumulation of GM1 gangliosides and other substances in the body’s lysosomes, causing GM1 gangliosidosis, a lysosomal storage disorder that results in progressive neurodegeneration and systemic complications.^[26]^ Interestingly, Ganglioside GM1 increased on senescent and aged endothelial cells. GM1 contributes to the impairment of insulin signaling in endothelial cells.^[27]^ Also, GM1 ganglioside is a marker of neurons and plays a key role in their differentiation, development, and regeneration. It stimulates the growth of nerve fibers, known as neurites, and accelerates their outgrowth.^[28]^ Thus, GLB1 may play key role in the development of DFU.

Our data showed that 2-linoleoylglycerol (18:2) mediated the GLB1 protein connected to DFU, also, 2 − linoleoylglycerol (18:2) levels showed a protective effect on DFU, and The odds ratio value is 0.4269 (95% CI: 0.2049–0.8893). There is a previous study showed that Type 2 diabetes patients showed a high 2 − linoleoylglycerol (18:2) levels than control. This discrepancy requires further investigation.

We recognize several limitations in our methodology. Although MR is a powerful approach for assessing causal relationships, its accuracy heavily relies on the strength and relevance of the selected IVs. Despite using rigorous selection criteria, residual confounding could still be a concern, particularly due to linkage disequilibrium or pleiotropic effects between the proteins and metabolites investigated. These confounding factors might affect the robustness of our results.

In addition to GLB1, 4 other proteins – AARSD1, EPHA2, TNFRSF10A, and IL7R – showed significant causal associations with DFU in our MR analysis. AARSD1 (alanyl-tRNA synthetase domain–containing 1) exhibited the strongest risk effect (OR = 18.91, 95% CI = 4.26–84.06). The specific mechanisms by which AARSD1 contributes to DFU remain unclear. AARSD1 appears to play context-dependent roles in various biological processes. For instance, in studies of chronic superficial gastritis, AARSD1 was identified among the genes with altered expression related to protein metabolism, suggesting its potential involvement in the regulation of protein biosynthesis, ubiquitination, and posttranslational modification.^[29]^ EPHA2, a member of the Eph receptor tyrosine kinase family, was positively associated with DFU risk (OR = 7.51, 95% CI = 2.52–22.42). EphA2 (EPH receptor A2) is a crucial receptor tyrosine kinase involved in cell-to-cell communication, guiding development, cell movement (migration, adhesion), and tissue structure.^[30]^ Its causal link with DFU highlights a possible shared pathway of microvascular dysfunction and impaired wound revascularization.

Tumor necrosis factor receptor superfamily member 10A (TNFRSF10A), also known as TRAIL-R1 or death receptor 4, is a cell surface protein that induces programmed cell death (apoptosis) upon binding with its ligand TRAIL. It plays a crucial role in immune responses, development, and cancer by mediating cell death or activating inflammatory pathways.^[31]^ In our study, TNFRSF10A showed a risk effect for DFU (OR = 1.68, 95% CI = 1.23–2.31). We speculate that TNFRSF10A may contribute to the development of DFU through apoptosis-related mechanisms. Finally, IL7R (interleukin-7 receptor) showed a moderate but significant risk association (OR = 1.46, 95% CI = 1.18–1.80), which is consistent with previous studies reporting that high glucose levels induce its upregulation – particularly in fibroblasts – thereby promoting ANGPTL4 secretion, inhibiting angiogenesis, and consequently impairing wound healing.^[32]^

In summary, our findings provide insights into the circulating proteome linked to DFU and highlight metabolites as crucial mediators. The mediation MR analysis further revealed that the GLB1 protein is associated with DFU through its effect on 2-linoleoylglycerol (18:2) levels.

## 5. Conclusion

In this MR study, we systematically explored the causal relationships between circulating proteins, metabolites, and DFU. Our analysis identified several key proteins – GLB1, AARSD1, EPHA2, TNFRSF10A, and IL7R – that exhibit significant causal associations with DFU risk. Among them, GLB1 demonstrated a protective effect mediated through the metabolite 2-linoleoylglycerol (18:2), suggesting a potential lipid-regulatory mechanism in DFU pathogenesis. In contrast, AARSD1, EPHA2, TNFRSF10A, and IL7R were identified as risk factors that may contribute to DFU through processes involving protein metabolism, vascular dysfunction, apoptosis, and impaired wound healing. Collectively, these findings reveal a complex molecular network linking circulating proteins and metabolites to DFU and highlight potential therapeutic targets for prevention and intervention. This study underscores the value of integrating multi-omics data with MR and mediation analysis to uncover biologically meaningful pathways in diabetic complications.

## Author contributions

**Conceptualization:** Minghao Wang, Shenghui Yu.

**Data curation:** Minghao Wang, Hu Yang.

**Formal analysis:** Minghao Wang, Lei Huang.

**Methodology:** Xin Hu.

**Software:** Lei Huang, Xin Hu, Hu Yang.

**Supervision:** Hu Yang.

**Validation:** Lei Huang.

**Visualization:** Shenghui Yu.

**Writing – original draft:** Minghao Wang, Hu Yang.





















## References

[1] McDermott K Fang M Boulton AJM Selvin E Hicks CW. Etiology, epidemiology, and disparities in the burden of diabetic foot ulcers. Diabetes Care. 2023;46:209–21. 36548709 10.2337/dci22-0043 PMC9797649

[2] Armstrong DG Tan T-W Boulton AJ Bus SA. Diabetic foot ulcers: a review. JAMA. 2023;330:62–75. 37395769 10.1001/jama.2023.10578 PMC10723802

[3] Chen L Sun S Gao Y Ran X. Global mortality of diabetic foot ulcer: a systematic review and meta-analysis of observational studies. Diabetes Obes Metab. 2023;25:36–45. 36054820 10.1111/dom.14840

[4] Yazdanpanah L Nasiri M Adarvishi S. Literature review on the management of diabetic foot ulcer. World J Diabetes. 2015;6:37–53. 25685277 10.4239/wjd.v6.i1.37 PMC4317316

[5] Navrazhina K Renert-Yuval Y Frew JW. Large-scale serum analysis identifies unique systemic biomarkers in psoriasis and hidradenitis suppurativa. Br J Dermatol. 2022;186:684–93. 34254293 10.1111/bjd.20642

[6] Del Duca E Renert-Yuval Y Pavel AB. Proteomic characterization of atopic dermatitis blood from infancy to adulthood. J Am Acad Dermatol. 2023;88:1083–93. 36773824 10.1016/j.jaad.2022.12.050 PMC10231669

[7] Geyer PE Hornburg D Pernemalm M. The circulating proteome─technological developments, current challenges, and future trends. J Proteome Res. 2024;23:5279–95. 39479990 10.1021/acs.jproteome.4c00586 PMC11629384

[8] Østergaard O Nielsen CT Iversen LV Jacobsen S Tanassi JT Heegaard NH. Quantitative proteome profiling of normal human circulating microparticles. J Proteome Res. 2012;11:2154–63. 22329422 10.1021/pr200901p

[9] Smith GD Ebrahim S. ‘Mendelian randomization’: can genetic epidemiology contribute to understanding environmental determinants of disease? Int J Epidemiol. 2003;32:1–22. 12689998 10.1093/ije/dyg070

[10] Sanderson E Glymour MM Holmes MV. Mendelian randomization. Nat Rev Methods Primers. 2022;2:6. 37325194 10.1038/s43586-021-00092-5 PMC7614635

[11] Emdin CA Khera AV Kathiresan S. Mendelian randomization. JAMA. 2017;318:1925–6. 29164242 10.1001/jama.2017.17219

[12] Smith GD Ebrahim S. Mendelian randomization: prospects, potentials, and limitations. Int J Epidemiol. 2004;33:30–42. 15075143 10.1093/ije/dyh132

[13] Yin K Qiao T Zhang Y. Unraveling shared risk factors for diabetic foot ulcer: a comprehensive Mendelian randomization analysis. BMJ Open Diabetes Res Care. 2023;11:e003523. 10.1136/bmjdrc-2023-003523 PMC10660165 37989345

[14] Bycroft C Freeman C Petkova D. The UK Biobank resource with deep phenotyping and genomic data. Nature. 2018;562:203–9. 30305743 10.1038/s41586-018-0579-z PMC6786975

[15] Allen N Sudlow C Downey P. UK biobank: current status and what it means for epidemiology. Health Pol Technol. 2012;1:123–6.

[16] Sun BB Chiou J Traylor M; Alnylam Human Genetics. Plasma proteomic associations with genetics and health in the UK Biobank. Nature. 2023;622:329–38. 37794186 10.1038/s41586-023-06592-6 PMC10567551

[17] Genomes Project C Auton A Brooks LD. A global reference for human genetic variation. Nature. 2015;526:68–74. 26432245 10.1038/nature15393 PMC4750478

[18] Lousdal ML. An introduction to instrumental variable assumptions, validation and estimation. Emerg Themes Epidemiol. 2018;15:1. 29387137 10.1186/s12982-018-0069-7 PMC5776781

[19] Glymour MM Tchetgen Tchetgen EJ Robins JM. Credible Mendelian randomization studies: approaches for evaluating the instrumental variable assumptions. Am J Epidemiol. 2012;175:332–9. 22247045 10.1093/aje/kwr323 PMC3366596

[20] Lawlor DA Harbord RM Sterne JA Timpson N Davey Smith G. Mendelian randomization: using genes as instruments for making causal inferences in epidemiology. Stat Med. 2008;27:1133–63. 17886233 10.1002/sim.3034

[21] Burgess S Thompson SG Collaboration CCG. Avoiding bias from weak instruments in Mendelian randomization studies. Int J Epidemiol. 2011;40:755–64. 21414999 10.1093/ije/dyr036

[22] Rasooly D Peloso GM. Two-sample multivariable mendelian randomization analysis using R. Curr Protocols. 2021;1:e335. 10.1002/cpz1.335 PMC8767787 34936225

[23] Mounier N Kutalik Z. Bias correction for inverse variance weighting Mendelian randomization. Genet Epidemiol. 2023;47:314–31. 37036286 10.1002/gepi.22522

[24] Aslam M. Cochran’s Q test for analyzing categorical data under uncertainty. J Big Data. 2023;10:147.

[25] Verbanck M Chen C-Y Neale B Do R. Detection of widespread horizontal pleiotropy in causal relationships inferred from Mendelian randomization between complex traits and diseases. Nat Genet. 2018;50:693–8. 29686387 10.1038/s41588-018-0099-7 PMC6083837

[26] Rha AK Maguire AS Martin DR. GM1 gangliosidosis: mechanisms and management. Appl Clin Genet. 2021;14:209–33. 33859490 10.2147/TACG.S206076 PMC8044076

[27] Sasaki N Itakura Y Toyoda M. Ganglioside GM1 contributes to the state of insulin resistance in senescent human arterial endothelial cells. J Biol Chem. 2015;290:25475–86. 26338710 10.1074/jbc.M115.684274 PMC4646194

[28] Guo Z. Ganglioside GM1 and the central nervous system. Int J Mol Sci. 2023;24:9558. 37298512 10.3390/ijms24119558 PMC10253378

[29] Yang ZM Chen WW Wang YF. [Study on gene differential expressions of substance and energy metabolism in chronic superficial gastritis patients of Pi deficiency syndrome and of pi-wei hygropyrexia syndrome]. Zhongguo Zhong Xi Yi Jie He Za Zhi. 2012;32:1180–7. 23185754

[30] Toracchio L Carrabotta M Mancarella C Morrione A Scotlandi K. EphA2 in cancer: molecular complexity and therapeutic opportunities. Int J Mol Sci. 2024;25:12191. 39596256 10.3390/ijms252212191 PMC11594831

[31] Kaczynski TJ Husami NJ Au ED Farkas MH. Dysregulation of a lncRNA within the TNFRSF10A locus activates cell death pathways. Cell Death Discov. 2023;9:242. 37443108 10.1038/s41420-023-01544-5 PMC10344863

[32] Gao R Zhou P Li Y Li Q. High glucose-induced IL-7/IL-7R upregulation of dermal fibroblasts inhibits angiogenesis in a paracrine way in delayed diabetic wound healing. J Cell Commun Signal. 2023;17:1023–38. 37217704 10.1007/s12079-023-00754-x PMC10409704

